# Daily stock index return for the Canadian, UK, and US equity markets, compiled by Morgan Stanley Capital International, obtained from Datastream

**DOI:** 10.1016/j.dib.2017.12.045

**Published:** 2017-12-21

**Authors:** Leon Li

**Affiliations:** School of Accounting, Finance and Economics, University of Waikato, New Zealand

## Abstract

The data presented in this article are related to the research article entitled “Testing and comparing the performance of dynamic variance and correlation models in value-at-risk estimation. North American Journal of Economics and Finance, 40, 116–135. doi:10.1016/j.najef.2017.02.006 (Li, 2017) [1]. Data on daily stock index return for the Canadian, UK, and US equity markets, as compiled by Morgan Stanley Capital International, are provided in this paper. The country indices comprise at least 80% of the stock market capitalization of each country. The data cover the period from January 1, 1990, through September 8, 2016, and include 6963 observations. All stock prices are stated in dollars.

**Specifications Table**

**Table:** 

Subject area	*Finance*
More specific subject area	*Estimation of market risk*
Type of data	*Table and figure*
How data was acquired	*Downloaded from database of Datastream*
Data format	*Raw*
Experimental factors	*NA*
Experimental features	*NA*
Data source location	*Canadian, UK, and US equity markets*
Data accessibility	*The data are available with this article and Datastream database*
Related research article	Li [1]

**Value of the data**

• The data is to test the performance of different models on estimation of the value of risk (VaR).
• VaR is one of the best single risk-measurement techniques. As such, VaR has been adopted by the Basel Committee to set the standard for the minimum amount of capital to be held against market risk.
• This data may allow researchers to test other research topics in finance, e.g., international portfolio management.

## 1 Data

**Fig. 1:**
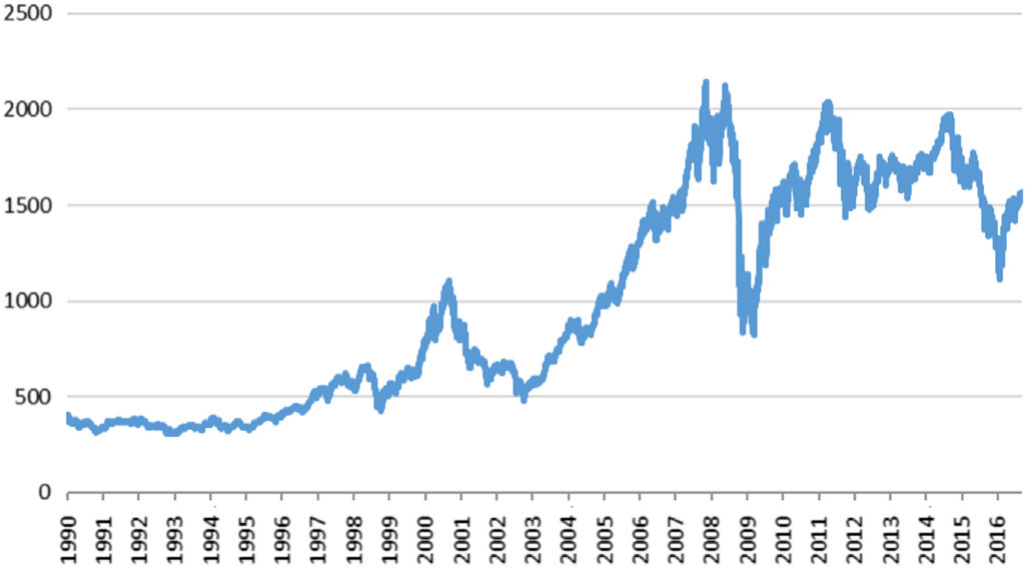
Daily stock index of Canada stock market.

**Fig. 2:**
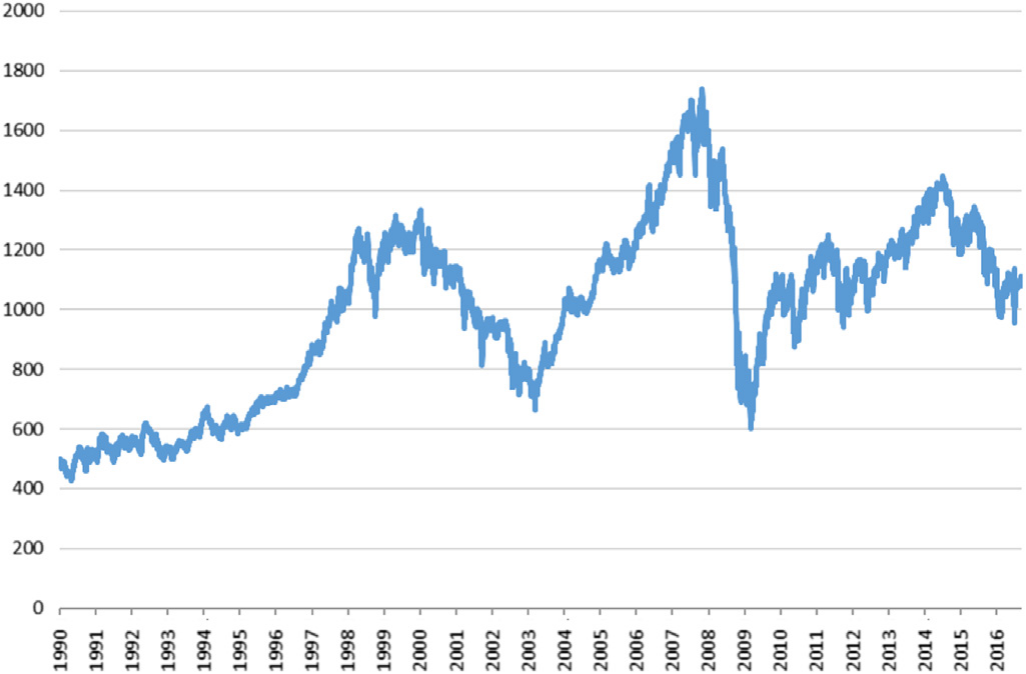
Daily stock index of UK stock market.

**Fig. 3:**
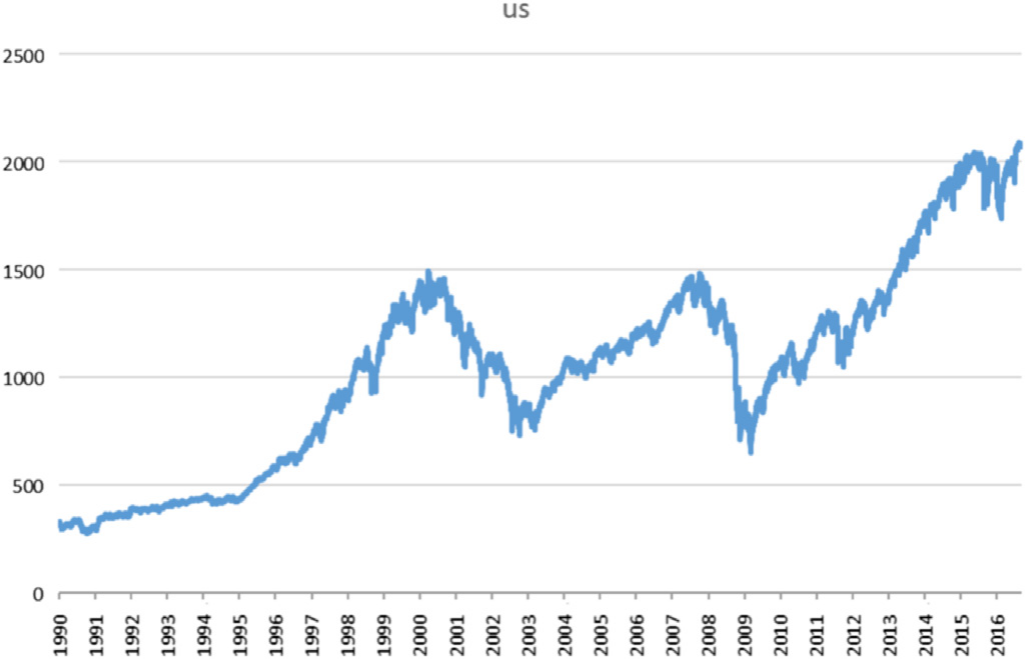
Daily stock index of US stock market.

The data contains the three daily stock index returns, including Canada, UK, and US, from 1990 to 2006. This investigation uses daily index returns. Fig. 1, Fig. 2, Fig. 3 present daily index of the three stock markets.

## 2 Experimental design, materials and methods

The data used in this paper is daily return rate of the three stock indices. I used OPTIMUM, a package program from GAUSS, and the built-in BFGS (Boyden, Fletcher, Goldfarb, and Shanno) algebra to derive the negative minimum likelihood (ML) function values of all the models. BFGS algebra is effective for deriving the maximum value of the non-linear likelihood functions. The mapped converged measure with the greatest ML function value then serves as the estimate.
